# Cocaine-regulated microRNA miR-124 controls poly (ADP-ribose) polymerase-1 expression in neuronal cells

**DOI:** 10.1038/s41598-020-68144-6

**Published:** 2020-07-08

**Authors:** Sabyasachi Dash, Muthukumar Balasubramaniam, Freddyson J. Martínez-Rivera, Arthur Godino, Emily G. Peck, Srinivas Patnaik, Mrutyunjay Suar, Erin S. Calipari, Eric J. Nestler, Fernando Villalta, Chandravanu Dash, Jui Pandhare

**Affiliations:** 1 Center for AIDS Health Disparities Research, Meharry Medical College, Old Hospital Bldg-CAHDR, Room 5023, 1005 Dr. DB Todd Jr Blvd., Nashville, TN 37208 USA; 2 Center for Molecular and Behavioral Neuroscience, Meharry Medical College, Nashville, TN 37208 USA; 3 School of Graduate Studies and Research, Meharry Medical College, Nashville, TN 37208 USA; 4 Department of Biochemistry, Cancer Biology, Pharmacology and Neuroscience, Meharry Medical College, Nashville, TN 37208 USA; 5 Department of Microbiology, Immunology, and Physiology, Meharry Medical College, Nashville, TN 37208 USA; 6 Nash Family Department of Neuroscience and Friedman Brain Institute, Icahn School of Medicine at Mount Sinai, New York, NY 10029 USA; 7 School of Biotechnology, Kalinga Institute of Industrial Technology University, Bhubaneswar, Odisha India; 8 Department of Pharmacology, Vanderbilt University Medical Center, Nashville, TN 37232 USA

**Keywords:** Cellular neuroscience, Molecular neuroscience, miRNAs, Biochemistry, Neuroscience

## Abstract

MiR-124 is a highly expressed miRNA in the brain and regulates genes involved in neuronal function. We report that miR-124 post-transcriptionally regulates PARP-1. We have identified a highly conserved binding site of miR-124 in the 3′-untranslated region (3′UTR) of *Parp-1* mRNA. We demonstrate that miR-124 directly binds to the *Parp-1* 3′UTR and mutations in the seed sequences abrogate binding between the two RNA molecules. Luciferase reporter assay revealed that miR-124 post-transcriptionally regulates *Parp-1* 3′UTR activity in a dopaminergic neuronal cell model. Interestingly, the binding region of miR-124 in *Parp-1* 3′UTR overlapped with the target sequence of miR-125b, another post-transcriptional regulator of *Parp-1*. Our results from titration and pull-down studies revealed that miR-124 binds to *Parp-1* 3′UTR with greater affinity and confers a dominant post-transcriptional inhibition compared to miR-125b. Interestingly, acute or chronic cocaine exposure downregulated miR-124 levels concomitant with upregulation of PARP-1 protein in dopaminergic-like neuronal cells in culture. Levels of miR-124 were also downregulated upon acute or chronic cocaine exposure in the mouse nucleus accumbens (NAc)-a key reward region of brain. Time-course studies revealed that cocaine treatment persistently downregulated miR-124 in NAc. Consistent with this finding, miR-124 expression was also significantly reduced in the NAc of animals conditioned for cocaine place preference. Collectively, these studies identify *Parp-1* as a direct target of miR-124 in neuronal cells, establish miR-124 as a cocaine-regulated miRNA in the mouse NAc, and highlight a novel pathway underlying the molecular effects of cocaine.

## Introduction

MicroRNAs (miRNAs) are a class of noncoding RNAs that post-transcriptionally regulate gene expression^1^. These regulatory RNAs are involved in almost every aspect of cellular biology including development, differentiation, proliferation, and death^2,3^. Accordingly, a number of miRNAs regulate gene expression during brain development, adult brain function, and neurological and psychiatric disorders^4^. Specifically, miRNAs such as miR-124, miR-132, miR-134, and miR-212 have been suggested to play critical roles in neuronal function, synaptic plasticity, and substance use disorders^5–7^.

Cocaine, a commonly used psychostimulant, has been reported to regulate a number of miRNAs in neuronal cells and in limbic brain reward regions^8–11^. For instance, cocaine treatment results in the upregulation of miR-134 and miR-135a in the hippocampus of rodent brain^10^ and miR-181a, miR-375, miR-212, and miR-132 in the striatum^9,12,13^. In addition, cocaine exposure has been shown to upregulate miR-9 levels in striatal post-synaptic densities^14^. Conversely, miR-183 levels were downregulated in the striatum upon cocaine treatment^14^. Consistent with these findings, a microarray study revealed that cocaine exposure resulted in the upregulation of ~ 25 miRNAs and downregulation of ~ 9 miRNAs in the hippocampus^10^. In addition to these miRNAs, miR-124 is emerging as a key cellular miRNA regulated by cocaine exposure in the central nervous system (CNS)^12,15–17^. miR-124 is highly expressed across the brain^18^ and is involved in neurogenesis, synaptic signal transmission, neurodegeneration, neuroadaptation, synapse morphology, long-term potentiation, and neuronal homeostasis^19–21^. First identified in mice^22^, miR-124 has been reported to regulate genes involved in the acquisition and maintenance of neuronal identity^23,24^. Decreased miR-124 levels have also been reported in several central nervous system (CNS) disorders including Parkinson’s disease, dementia, multiple sclerosis, and substance use disorders^25–27^. Since these studies establish the importance of miR-124 in the brain, understanding the role of this miR will provide key insights into the molecular actions of cocaine.

Regulation of miR-124 by cocaine was first described in a rodent model through a miRNA profiling study^12^. Subsequently, it was reported that downregulation of miR-124 influences cocaine-induced synaptic plasticity and behavioral changes^17^. Accordingly, overexpression of miR-124 in the nucleus accumbens (NAc), a key brain reward region, resulted in the attenuation of cocaine-induced conditioned place preference (CPP)^17^. Recently, cocaine treatment has been shown to downregulate miR-124 expression in neuroblastoma cells^15^. In addition, published studies indicate that miR-124 regulates key genes involved in brain function^16, 28^. These include genes involved in neurogenesis such as *Scp1,* *Baf53a,* *Ptbp1,* *RhoG,* *Jagged1*, and *Sox9*^19^. miR-124 has also been reported to regulate *Nr3c1* and *Gria4*, which are associated with glutamate signaling^29^ and *Bdnf* and *Drd3*, genes which are directly implicated in cocaine-induced plasticity^17^. Additionally, miR-124 has been suggested to suppress a network of non-neuronal genes to promote neuronal identity^23,24^. Although these studies identify several genes regulated by miR-124, the mechanism by which miR-124 regulates the molecular effects of cocaine remains largely speculative.

To identify key gene(s) regulated by miR-124 during cocaine exposure, first we carried out in silico studies that predicted a highly conserved binding site of miR-124 within the 3′UTR sequences of *Parp-1*. PARP-1 is a DNA repair enzyme^30^, and PARylates a number of target proteins^31–33^. Therefore, it has been implicated in a wide range of biological processes^34^ including neuronal function^35–38^. For example, PARP-1 is important for growth of axons^39^, neuronal responses^40^, neuronal dysfunction and death^41^. Most importantly, PARP-1 and PARylation have been shown to be involved in the molecular effects of cocaine in the NAc^42–44^. Given these important roles of PARP-1, we tested the functional significance of our in silico prediction studies in both a dopaminergic neuronal cell model and rodent brain. Genetic manipulation, luciferase reporter, and pull-down assays in the neuronal cell model demonstrated that miR-124 post-transcriptionally regulates *Parp-1* by directly binding to its 3′UTR. Importantly, acute or chronic cocaine exposure downregulated miR-124 concomitant with upregulation of PARP-1 expression in the mouse NAc. Finally, miR-124 levels were also significantly reduced in the NAc of animals after induction of conditional place preference (CPP) for cocaine. Collectively, these studies identify *Parp-1* as a direct target of miR-124 in neuronal cells and unravel a novel regulatory mechanism underlying the molecular effects of cocaine exposure.

## Results

### miR-124 is a post-transcriptional regulator of Parp-1 in neuronal cells

**Figure 1:**
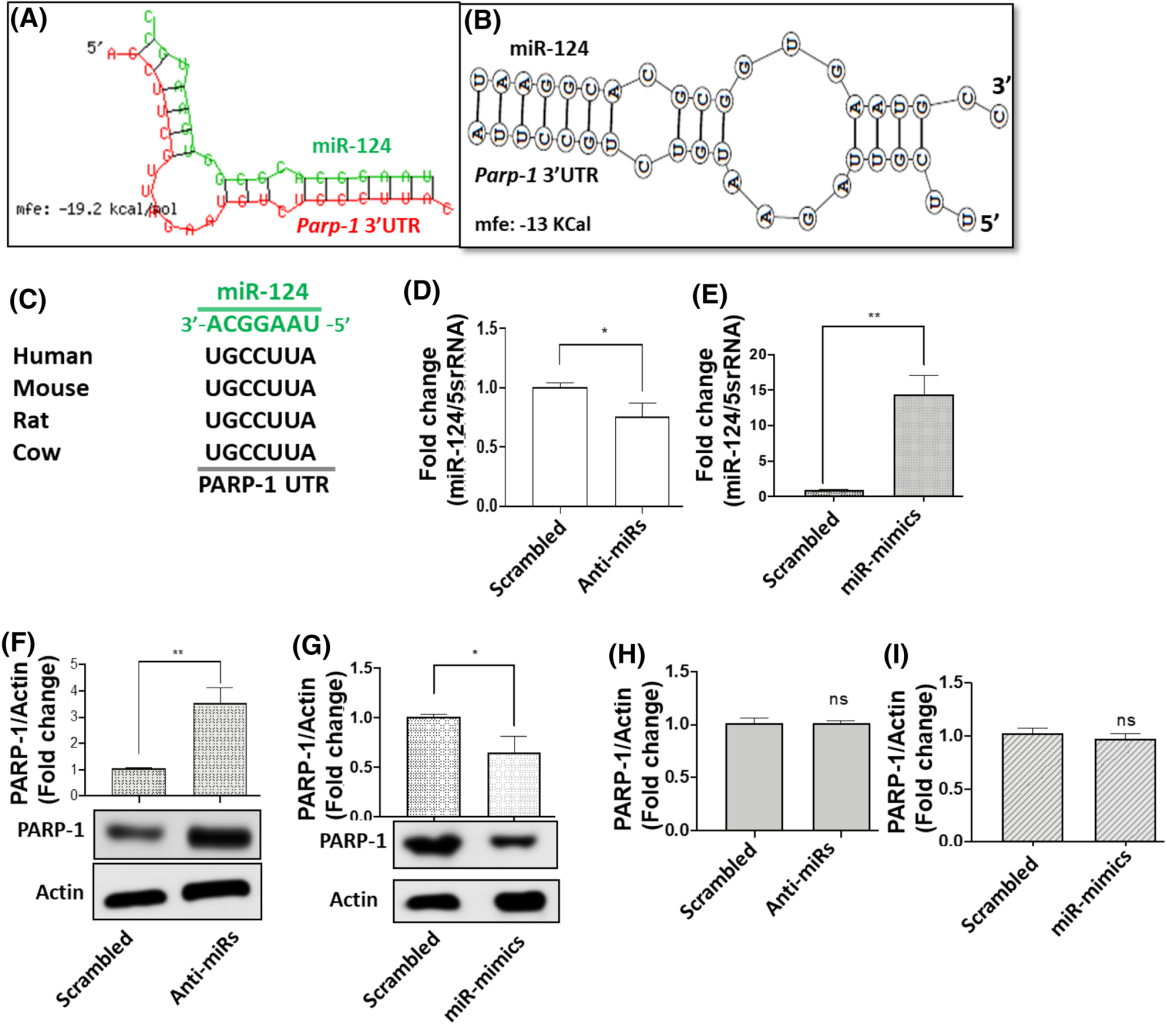
miR-124 negatively regulates PARP-1 expression. (**A**–**C**) In silico* analysis of Parp-1 3′UTR*. (**A**) RNAHybrid2.1 analysis and (**B**) BiFold:RNA Structures analysis predicted a putative binding site of miR-124 in the 3′UTR of *Parp-1* and the formation of a hair-pin structure between the two RNA molecules. (**C**) Sequence alignment of miR-124 binding site in the 3′UTR of *Parp-1* among different mammalian species. (**D**–**G**) *Effects of miR-124 expression on PARP-1 protein levels*. For knockdown and overexpression studies, anti-miRs or miR-mimics were transfected into differentiated SH-SY5Y cells and 24 h post-transfection, levels of miR-124 was measured by qPCR. (**D**) Knockdown studies show dramatic reduction in miR-124 levels, whereas (*E*) Overexpression studies show marked increases in miR-124 expression. (**F**) PARP-1 protein expression in miR-124 knocked down cells, and (**G**) miR-124 overexpressing cells was measured by immunoblot. (**H**, **I**) Effects of miR-124 expression on Parp-1 mRNA levels. *Parp-1* mRNA expression in (**H**) miR-124 knockdown and (**I**) miR-124 overexpressing cells was measured by qPCR. Data presented in **D**–**I** are mean values of at least three independent experiments conducted in triplicates with error bars representing the standard error of the mean (± SEM). *represents *p* < 0.05, whereas **represents *p* < 0.005 for the comparison of anti-miR/miR-mimics vs scrambled controls. The immunoblot data in **F**, **G** (lower panel) are representative blots from at least (n = 3) independent experiments.

To better understand the role of miR-124 during cocaine exposure, we carried out in silico analysis to identify target genes of this miR. We used two independent algorithms, RNAhybrid 2.1^45, 46^ and biFold:RNA Structures^47^ to enhance confidence in the prediction accuracy. Interestingly, both of these in silico platforms predicted a miR-124 binding site in the 3′UTR of *Parp-1* mRNA (Fig. 1A-C). These studies also depicted the formation of a hairpin structure between the two RNA molecules (Fig. 1A-B). The miR-124 target site was located at the nucleotide position 312–331 of *Parp-1* 3′UTR (Fig. 1C) and was highly conserved in several mammalian species (Fig. 1C), another critical feature of miRNA-mediated gene regulation^2^.

To probe the biological relevance of the predicted binding site, we tested whether miR-124 regulates PARP-1 expression in neuronal cells. We carried out knockdown and overexpression studies in differentiated SH-SY5Y neuroblastoma cells since these cells express several markers of a dopaminergic neuronal phenotype and endogenously express PARP-1^48^. qPCR data presented in Fig. 1D show that cells transfected with the anti-miRs contained significantly lower miR-124 levels compared to the control cells. Similarly, miR-124 levels were markedly enhanced in the cells transfected with the miR-mimics (Fig. 1E). Concurrently, measurement of PARP-1 protein levels by Western blot show that cells transfected with anti-miRs express higher levels of PARP-1 protein compared to the control cells (Fig. 1F). Conversely, higher miR-124 levels led to a significant down-regulation of PARP-1 protein levels (Fig. 1G). Since miRNAs can sometimes affect protein expression by reducing mRNA levels^23, 49^, we probed the effects of miR-124 on *Parp-1* mRNA levels. qPCR analysis showed only minimal changes in *Parp-1* mRNA levels when miR-124 was knocked down (Fig. 1H) or overexpressed (Fig. 1I). Collectively, these results demonstrate a negative association between miR-124 and PARP-1 protein levels and strongly suggest a post-transcriptional mechanism of PARP-1 regulation by miR-124 in neuronal cells.

### miR-124 directly binds to the Parp-1 3′-UTR

**Figure 2:**
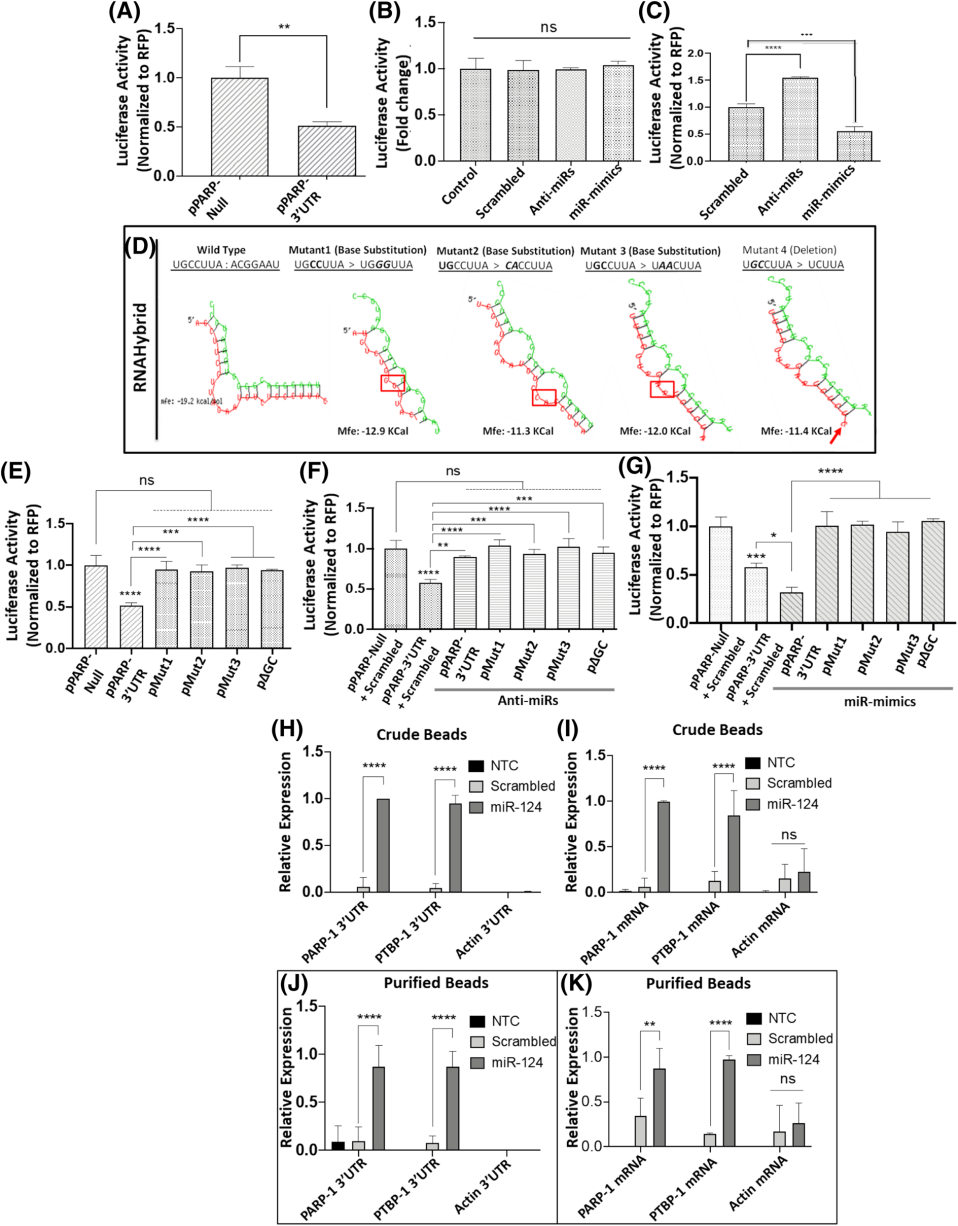
miR-124 targets the 3′UTR of *Parp-1* for post-transcriptional regulation. (**A**–**C**) *Effects of miR-124 expression on Parp-1 3′UTR activity*. (**A**) Luciferase reporter constructs without the 3′UTR of *Parp-1* (pPARP-Null) or with the 3′UTR (pPARP-3′UTR) were transfected into differentiated SH-SY5Y cells. Luciferase activity in the cellular lysates was measured after 24 h post transfection. (**B**) pPARP-Null reporter was co-transfected with either scrambled controls or anti-miR-124 or miR-124 mimics into differentiated SH-SY5Y cells and luciferase activity was measured. (**C**) Luciferase activity of lysates prepared from differentiated SH-SY5Y cells co-transfected with pPARP-3′UTR with either scrambled controls or anti-miR-124 or miR-124 mimics. (**D**) In silico* analysis of seed sequence mutations on miR-124 and Parp-1 3′UTR interaction*. We introduced four different mutations [(Mutant1: CC > GG), (Mutant2: TG > CA), (Mutant3: GC > AA), and (Mutant4: ΔGC with GC deletion)] in the miR-124 binding site of the *Parp-1* 3′UTR. The effects of these mutations on the stability (as measured by MFE) and secondary hair-pin loop structure was analyzed by RNAhybrid 2.1. (**E**–**G**) *Effects of seed sequence mutations on Parp-1 3′UTR activity*. Luciferase assay was employed to measure the effects of seed sequence mutation on *Parp-1* 3′UTR activity. Reporter constructs containing the mutations [(pMut1 with CC > GG), (pMut2 with TG > CA), (pMut3 with GC > AA), and pΔGC with GC deletion) were generated by site-directed mutagenesis. (**E**) Luciferase activity in the lysates of differentiated SH-SY5Y cells transfected with pPARP-Null, pPARP-1 3′UTR, and mutant pPARP-1 3′UTRs. Luciferase activity of lysates prepared from cells co-transfected with pPARP-Null, pPARP-1 3′UTR, and mutant pPARP-1 3′UTRs in the presence or absence of (**F**) anti-miR-124 and, in the presence of (**G**) miR-124 mimics. (**H**–**K**) For pull-down assays, LNA-based biotinylated-miR-124 mimics or scrambled controls were transfected into differentiated SH-SY5Y cells. Enrichment of miR-124:mRNA complexes were quantified by qPCR. To detect the target mRNAs two sets of primers were used. One set targeted the 3′UTR region ORF (**H** and **J**) and the second set amplified the ORF region (**I** and **K**). *Ptbp-1* was used as a positive control and *β-actin* as the negative control. Black bars indicate target expression in no template controls (NTC), light bars represent qPCR data of pull-downs with scrambled controls whereas dark grey bars represent qPCR data of pull-downs with miR-mimics. Data presented in **A**–**C**, **E**–**G**, and **H**–**K** are mean values of (n = 3) independent experiments conducted in triplicates with error bars representing ± SEM. *represents *p* < 0.05, **p < 0.005, ***p < 0.001, and ****p < 0.0005 for statistical significance, whereas ns represents not significant.

Since miRNAs negatively regulate protein expression by targeting the 3′-UTR of the target mRNA^50,51^, we probed the interaction between miR-124 and the 3′-UTR sequences of *Parp-1* mRNA. We employed a luciferase reporter assay using a vector containing the 3′-UTR of *Parp-1* that is cloned downstream of the luciferase gene^48^. Cells transfected with the pPARP-1 3′UTR showed lower luciferase activity when compared to the pPARP-Null control plasmid (Fig. 2A). We next carried out co-transfection studies of pPARP-Null (Fig. 2B) or pPARP-3′UTR (Fig. 2C) in the presence or absence of miR-mimics or anti-miRs. Data in Fig. 2B show that altering the levels of miR-124 did not affect luciferase activity in the cells transfected with the pPARP-Null control plasmid. However, knockdown of miR-124 using anti-miR resulted in significant induction of *Parp-1* 3′UTR driven luciferase reporter activity (Fig. 2C). Conversely, increasing miR-124 levels by transfecting miR-mimics resulted in reduced *Parp-1* 3′UTR driven luciferase activity (Fig. 2C) but not that of the *Parp-1* Null. Collectively, these results suggest that *Parp-1* 3′-UTR activity is regulated by miR-124 expression in neuronal cells.

To further characterize the interaction between miR-124 and *Parp-1* 3′UTR, we carried out site-directed mutagenesis studies. First, we performed in silico mutational analyses by introducing three different substitutions [CC > GG (pMut1), TG > CA (pMut2), GC > AA (pMut3)] and one deletion [ΔGC (pΔGC)] in the 3′-UTR (Fig. 2D). Structural models with lowest minimum free energy (MFE) revealed that these mutations resulted in reduced stability and binding affinity between the *Parp-1* 3′UTR and miR-124 (Fig. 2D). Then, we tested the effects of these mutations on miR-124 interaction by luciferase reporter assay. As expected lower luciferase activity was observed in cells transfected with pPARP-1 3′UTR alone when compared to pPARP-Null control (Fig. 2E). To specifically study the effects of miR-124 on PARP-1 3′UTR driven luciferase activity, we co-transfected anti-miRs or miR-mimics with the control plasmid (pPARP-Null), or plasmid harboring *Parp-1* 3′UTR (pPARP-1 3′UTR) or the mutant plasmids into differentiated SH-SY5Y cells. As expected, overexpression of miR-124 reduced luciferase activity driven by *Parp-1* 3′UTR relative to the control (Fig. 2G). However, in cells transfected with the mutant plasmids, higher levels of miR-124 showed minimal effect on luciferase activity (Fig. 2G). Accordingly, knockdown of endogenous miR-124 levels resulted in a significant enhancement of luciferase activity (Fig. 2F). In contrast, transfection of *Parp-1* 3′UTR mutants did not enhance luciferase activity (Fig. 2F). Collectively, these studies demonstrate that miR-124 directly interacts with *Parp-1* 3′UTR by binding to its seed sequence.

### Biotinylated-miR-124 pulls-down Parp-1 mRNA

To further probe direct binding between miR-124 and *Parp-1* 3′UTR, we employed a pulldown assay using biotinylated LNA-based miR-124 mimics^48^. The miR-124 mimics or scrambled controls were transfected into differentiated SH-SY5Y cells and the miR-124-*Parp-1* complex was pulled down with streptavidin-coated magnetic beads. Then, *Parp-1* mRNA was quantified by qPCR using two sets of primers; the first set targeted a region in the ORF and the second set amplified the 3′UTR. *Ptbp-1* was used as a positive control since it is an established target of miR-124^52^, and *β-actin* which lacks miR-124 binding sequences served as the negative control. qPCR analyses of both the crude and purified beads show that *Parp-1* mRNA was highly enriched in the pull-down of crude and purified samples (Fig. 2H and J). Amplification of the 3′UTR regions of the target genes in the crude and purified beads also showed enrichment of both *Parp-1* 3′UTR and *Ptbp-1* 3′UTR in the biotin-tagged miR-124 mimic assisted pull-downs (Fig. 2I and K). Collectively, these data provide further evidence that miR-124 directly binds to *Parp-1* mRNA to negatively regulate PARP-1 protein expression.

### miR-124 has a dominant regulatory effect on Parp-1 3′-UTR compared to miR-125b

**Figure 3:**
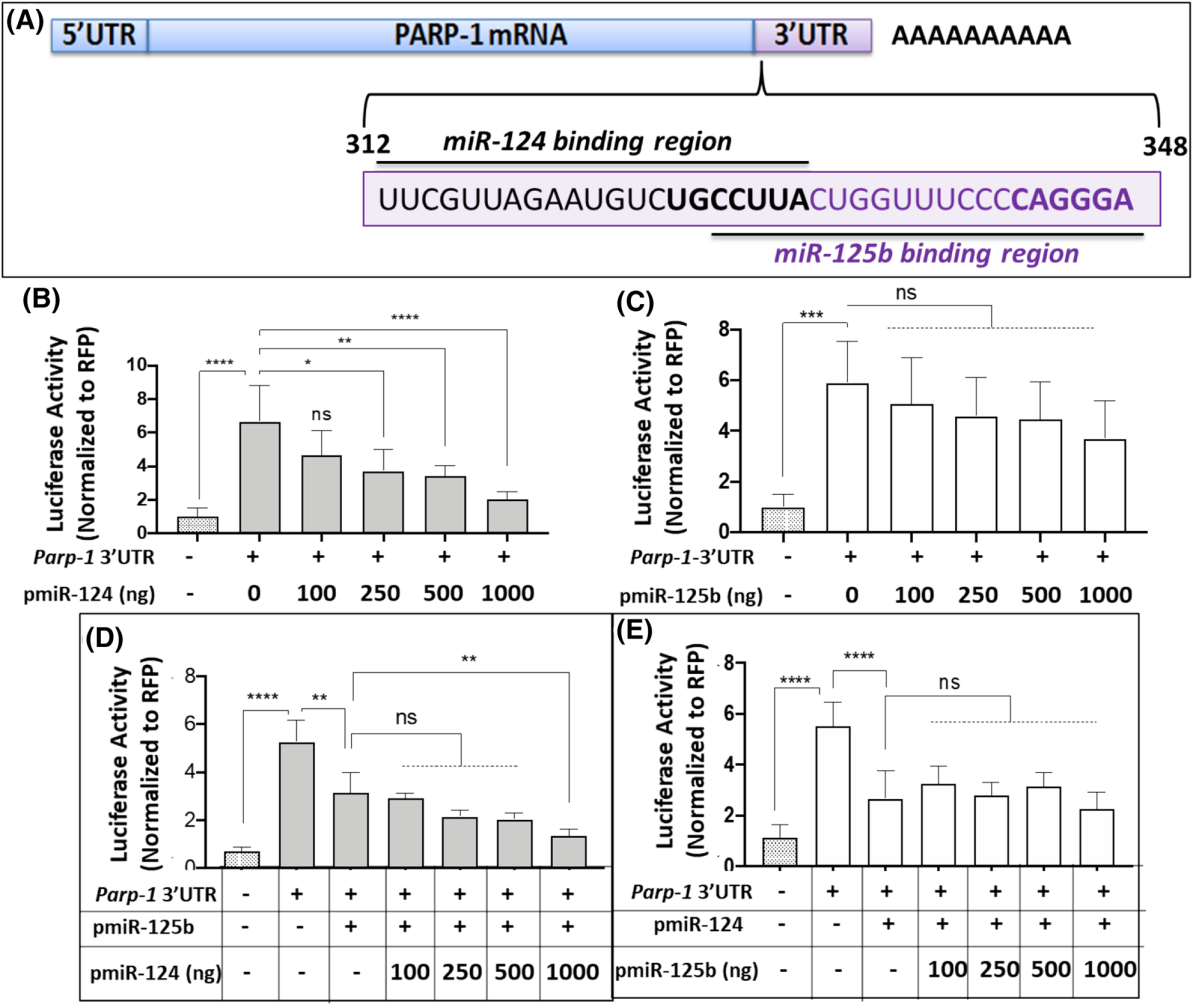
miR-124 confers greater regulatory activity on *Parp-1* 3′UTR compared to miR-125b. (**A**) Schematic representation of *Parp-1 *mRNA showing the overlapping binding regions of miR-124 and miR-125b in the 3′UTR sequences. Dose-dependent effects of (**B**) miR-124 and (**C**) miR-125b on *Parp-1 *3′UTR-driven luciferase activity. Luciferase activity of lysates prepared from differentiated SH-SY5Y cells co-transfected with pPARP-3′UTR and increased amounts of (**B**) pmiR-124 and (**C**) pmiR-125b. Effects of titrating (**D**) miR-124 against miR-125b and (**E**) miR-125b against miR-124 on *Parp-1 *3′UTR activity (**D**) HEK-293 T cells were co-transfected with *Parp-1 *3′UTR and pmiR-125b, in the absence of presence of increase amounts of pmiR-124. 24 h post transfection, cellular lysates were prepared and luciferase activity was measured. (**E**) Luciferase activity of lysates prepared from HEK-293 T cells co-transfected with *Parp-1 *3′UTR and pmiR-124, in the absence of presence of increase amounts of pmiR-125b. Data presented in (**B**–**E**) are mean values of (n = 3) independent experiments conducted in triplicates with error bars representing the ± SEM. *represents *p* < 0.05, **p < 0.005, ***p < 0.001, and ****p < 0.0005 for statistical significance, whereas ns represents not significant.

We have previously reported that the cellular miRNA miR-125b post-transcriptionally regulates *Parp-1* in neuronal cells^48^. Notably, the binding region of miR-124 in *Parp-1* 3′UTR significantly overlapped with that of miR-125b (Fig. 3A). Specifically, the seed sequence for miR-125b is located 10 nucleotides downstream of the miR-124 seed sequence (Fig. 3A). Since multiple miRNAs can regulate a single gene^1^, the overlapping binding region suggested that post-transcriptional regulation of PARP-1 could be achieved by both miR-124 and miR-125b. To test this prediction, we titrated miR-124 and miR-125b against each other and measured *Parp-1* 3′UTR-driven luciferase activity. As expected, increased expression of miR-124 resulted in a dose dependent decrease in *Parp-1* 3′UTR-driven luciferase activity (Fig. 3B). A dose-dependent effect of miR-125b was also observed on *Parp-1* 3′UTR activity (Fig. 3C). At the initial concentration of 100 ng of miR-124 (Fig. 3B) and miR-125 levels (Fig. 3C), a minimal inhibition in 3′UTR driven luciferase activity was observed. However, a significant reduction in luciferase activity was observed only for increasing amounts of miR-124 (Fig. 3B) but not for miR-125b titrations (Fig. 3C). Then, we performed titration experiments through co-transfections of pPARP-3′UTR with pmiR-124 and pmiR-125b. Data shown in Fig. 3D indicate that titrating an increasing amount of miR-124 in a background of a specific amount of miR-125b (250 ng of pmiR-125b) resulted in a dose-dependent reduction in luciferase activity suggesting that miR-124 can assert inhibitory effects on PARP-1 translation even in the presence of miR-125b (Fig. 3D). Surprisingly, when increasing amounts of pmiR-125b were titrated in the presence of pmiR-124 (250 ng), there was only a minor reduction in luciferase activity when compared to pmiR-124 alone (Fig. 3E). These comparative analyses indicate that, in the presence of miR-124, the effects of miR-125b on *Parp-1* UTR is reduced, suggesting a dominant post-transcriptional regulation of PARP-1 by miR-124.

### miR-124 expression is reduced concurrent with increased PARP-1 upon acute or chronic cocaine treatment in differentiated dopaminergic neuronal-like cells

**Figure 4:**
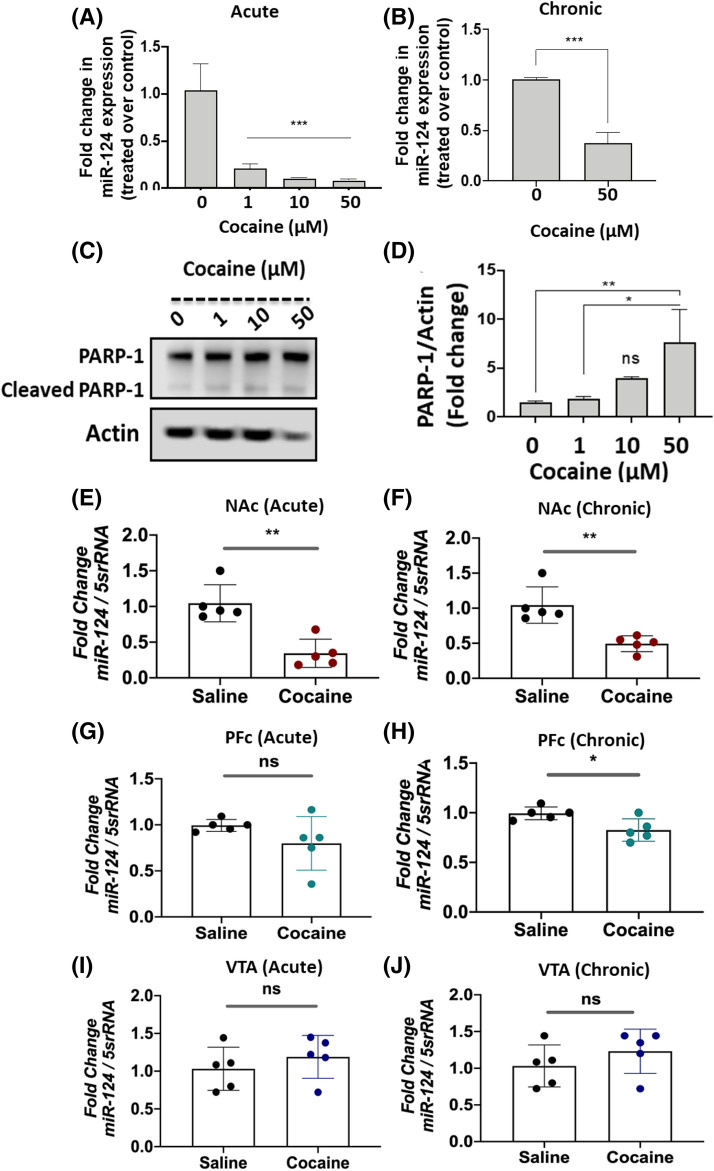
Cocaine exposure downregulates miR-124 expression in dopaminergic neuronal cell model and in the NAc of rodent brain. **(A**–**D)** SH-SY5Y cells were cultured overnight and then differentiated for 5 days in presence of Retinoic acid. **A**, **B** *Effects of cocaine exposure on miR-124 expression*. (**A**) Differentiated cells were treated with varying concentrations of cocaine (0–50 µM) overnight for acute exposure. (**B**) For chronic treatment, differentiated cells were treated with cocaine at 50 µM for 5 days (once every 24 h). Thereafter, miRNAs were isolated from these cells and miR-124 expression was analyzed by qPCR. miR-124 expression was measured relative to the expression of 5S rRNA levels and relative expression of miR-124 in cocaine-treated cells compared to the untreated control cells. (**C,** **D**) *Effects of cocaine treatment on PARP-1 expression*. Differentiated SH-SY5Y cells were treated with varying concentrations of cocaine under acute conditions. Cells were then harvested and cellular lysates were subjected to immunoblot analysis. (**C**) Representative immunoblot of PARP-1 expression (n = 3). (**D**) Densitometry analysis of total PARP-1 expression normalized to actin. (**E–J**) *Acute and chronic cocaine exposure downregulates miR-124 in the mouse NAc*. Mice in each treatment group (n = 5) were injected intra-peritoneal (IP) with a single dose of cocaine (20 mg/kg) for acute cocaine treatment and at a dose of 20 mg/kg daily for 5 days for chronic exposures. Then animals were euthanized and NAc, PFC, and VTA were isolated from cocaine treated (n = 5) and saline-treated animals (n = 5). miRNAs isolated from the tissue samples were subjected to qPCR. miR-124 expression was measured relative to the expression of 5 s rRNA levels. (**E, F**) Relative expression of miR-124 in the NAc of cocaine-treated mice compared to the NAc of saline-treated mice in (**E**) acute and (**F**) chronic conditions. (**G**, **H**) miR-124 expression in the PFC of animals exposed to cocaine under (**G**) acute and (**H**) chronic conditions (**I**, **J**) miR-124 levels in the VTA of animals under (**I**) acute and (**J**) chronic cocaine treatment. Data presented are mean values of three independent experiments conducted in triplicates with error bars representing SEM. *represents *p* < 0.05, **p < 0.005, and ***p < 0.001 for statistical significance, whereas ns represents not significant.

Next, we investigated whether cocaine treatment altered miR-124 expression in neuronal cells. SH-SY5Y cells were differentiated into a dopaminergic neuronal-like phenotype as demonstrated by the expression of the dopamine transporter (DAT; the protein target of cocaine) and tyrosine hydroxylase (TH; the rate limiting enzyme in dopamine biosynthesis) (46). These cells were treated with increasing concentrations (0–50 µM) of cocaine mimicking acute or chronic treatment conditions. qPCR analysis revealed a dose dependent reduction in miR-124 levels in cells treated with cocaine under acute conditions relative to the untreated cells (Fig. 4A). For instance, miR-124 expression was reduced by ~ 60% with 1 µM cocaine (Fig. 4A), followed by an even dramatic reduction in miR-124 expression with increasing drug doses (Fig. 4A). Treatment with 50 µM cocaine resulted in a fivefold decrease in miR-124 levels. Similarly, chronic cocaine treatment with 50 µM also led to a significant downregulation of miR-124 expression (Fig. 4B). These results establish that cocaine treatment significantly down-regulates miR-124 expression in dopaminergic neuronal-like cells.

Since results in Figs. 1 and 2 showed that miR-124 post-transcriptionally regulates PARP-1, we investigated whether cocaine-induced downregulation of miR-124 is associated with altered PARP-1 expression in neuronal-like cells. As expected, a dose-dependent increase in total PARP-1 levels was observed with cocaine treatment (Fig. 4C). Densitometry analysis showed a significant increase in total PARP-1 expression in cells treated with 50 µM cocaine when compared to untreated cells (Fig. 4D). Interestingly, cocaine treatment did not increase the cleavage of PARP-1 (Fig. 4C) that serves as an indicator of cellular apoptosis^53^. The lack of PARP-1 cleavage is consistent with previously published studies showing that exposure to physiologically relevant concentrations (up to 100 µM) of cocaine does not induce significant neuronal apoptosis^48^. Collectively, these data indicate that cocaine-induced downregulation of miR-124 is inversely correlated with increased PARP-1 expression in neuronal-like cells.

### Acute or chronic cocaine treatment reduces miR-124 expression in the mouse NAc

To probe the in vivo significance of miR-124 regulation by cocaine, we examined miR-124 expression in mouse NAc, a crucial brain reward region. Data in Fig. 4E reveal that acute cocaine treatment resulted in a significant reduction in miR-124 expression (~ 60%) in the NAc when compared to saline controls. miR-124 expression was also dramatically reduced (~ 60%) in the NAc of animals exposed to chronic cocaine (Fig. 4F). In contrast, acute cocaine treatments did not affect the levels of miR-124 expression in the prefrontal cortex (PFC) (Fig. 4G). However, a reduction of ~ 20% for miR-124 levels was detected in the PFC after chronic cocaine treatments (Fig. 4H). Interestingly, in the ventral tegmental area (VTA), a midbrain dopaminergic nucleus that innervates the NAc, miR-124 levels were significantly elevated by acute or chronic cocaine treatments (Fig. 4I and J).

**Figure 5:**
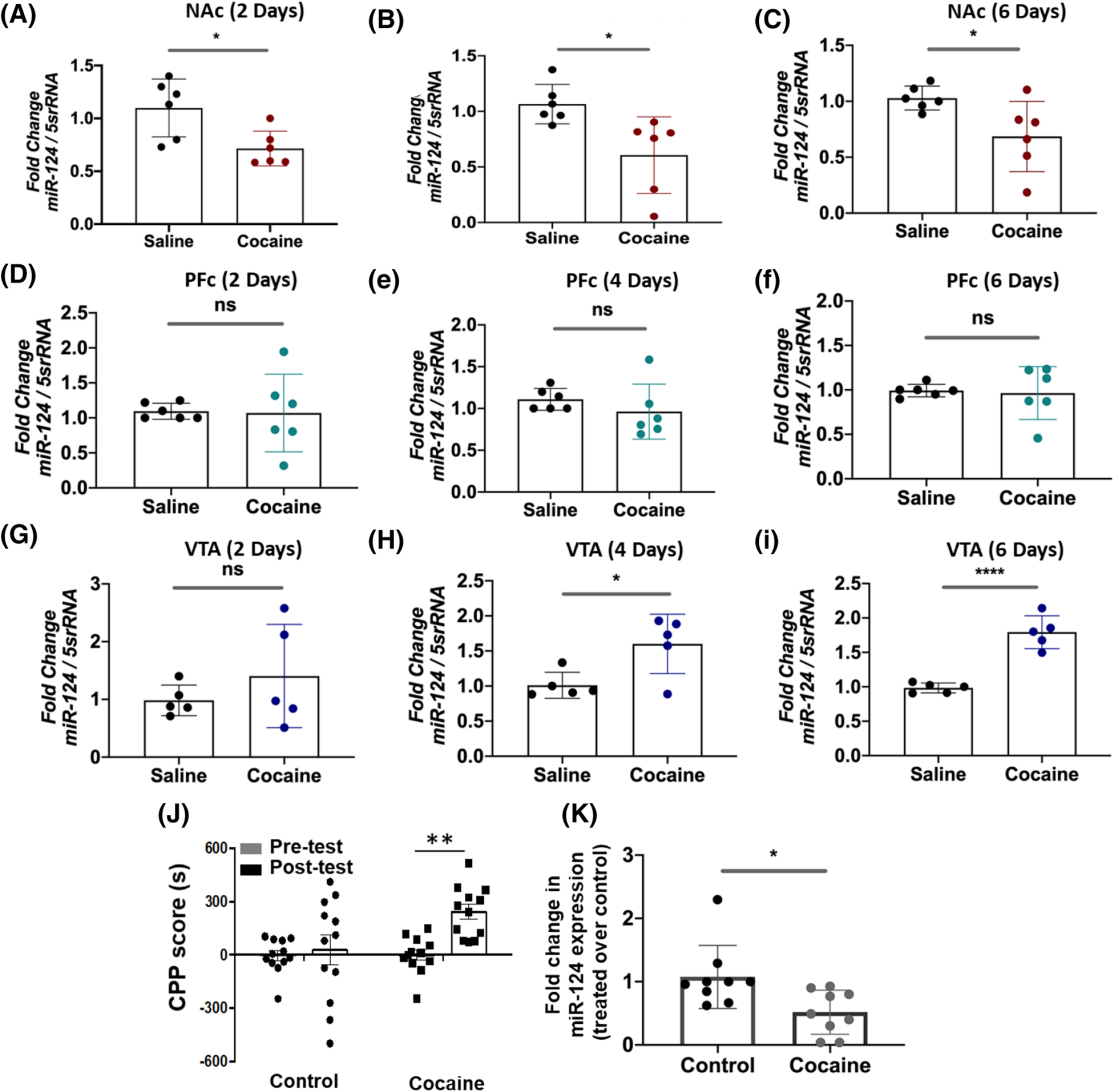
Time-dependent and conditioned for place preference effects of cocaine exposure on miR-124 expression in the reward circuitry of mice brain. (**A**–**I**) *Time-dependent effects of cocaine exposure on miR-124*. Mice were injected with a single dose of cocaine (20 mg/kg) daily until Day 6. On the day 2, 4, and 6, mice were euthanized, NAc, PFC, and VTA were used for miRNA isolations. miR-124 expression was measured by qPCR and expressed as relative to the expression of 5 s rRNA levels. Relative expression of miR-124 in the respective tissue regions of cocaine-treated mice (n = 6) were compared to that of saline-treated mice (n = 6) for each time point. Effects of cocaine on miR-124 expression levels at day 2 (**A**); day 4 (**B**); and day 6 (**C**) in the NAc. Time-dependent effects of cocaine on miR-124 expression in the PFC at day 2 (**D**), day 4 (**E**), and day 6 (**F**). miR-124 expression levels in the VTA of mice brain at day 2 (**G**); day4 (**H**); and day 6 (**I**). Data in (**A–I**) are average normalized expression values from n = 6, per experimental group for each time point, conducted in triplicates with error bars representing ± SEM. (**J**, **K**) Cocaine-induced CPP of mice. (**J**) CPP scores were calculated as time spent by the animals (n = 12 for cocaine-treated group and n = 12 for saline-treated group) in the cocaine-paired chamber (20 mg/kg i.p.) minus time spent in the saline-paired chamber. On day 8, mice were euthanized, NAc tissue was used for miRNA isolations from cocaine and saline treated groups. miR-124 expression was measured by qPCR and expressed as relative to the expression of 5 s rRNA levels. **K **Expression of miR-124 of cocaine-treated mice (n = 9) were plotted relative to that of saline-treated mice (n = 9). Paired two-tailed *t* test was used to compare the pre-test vs. post-test independently within control and cocaine groups in panel **J**. CPP score for animals conditioned for cocaine (day8 post-test vs. day1 pre-test) whereas Unpaired two-tailed *t* test was employed to determine significance of difference in miR-124 levels between saline and cocaine treatments in Panel **K**. Data from the qPCR experiments are average normalized expression values from n = 9, per group, conducted in triplicates with error bars representing ± SEM. *represents *p* < 0.05, and ***p < 0.001 for statistical significance, whereas ns represents not significant.

We next carried out time-course studies to further understand the effects of cocaine exposure on miR-124 expression in the mouse brain. Data from these analyses show that miR-124 levels in the NAc were reduced up to ~ 40% by day 2 (Fig. 5A) and remained lower by day 4 (Fig. 5B) and day 6 upon cocaine treatment when compared to respective saline controls (Fig. 5C). In contrast, there was a minimal to no change in miR-124 expression in the PFC even by day 6 (Fig. 5D–F). However, miR-124 expression in the VTA was induced from day 4 with subsequent induction through day 6 of cocaine treatment relative to the untreated controls (Fig. 5G–I). Collectively, these results show that cocaine reduces miR-124 expression specifically in the NAc with a modest effect in the PFC (under chronic exposure) and induces the expression of this miRNA in the VTA.

### miR-124 expression is reduced in the NAc of mice after conditioning for cocaine place preference

Our results showed that cocaine persistently downregulates miR-124 levels in the NAc of mice after acute or chronic exposure (Figs. 4, 5). To examine whether miR-124 levels are altered during cocaine associated behavioral effects, cocaine-induced conditioned place preference (CPP) experiments were conducted. Mice were conditioned to cocaine in a 6-day CPP protocol^54,55^ and compared with mice receiving saline as control. As expected, animals conditioned for cocaine showed a significantly higher CPP score on day 8 (Post-test) relative to day 1 (Pre-test) or when compared to the control mice at day 8 (Fig. 5J). On the test day after measurement of CPP the mice were immediately euthanized and punches of the NAc were flash frozen for RNA isolation and qPCR analyses. miR-124 expression levels showed a significant downregulation in the NAc in animals conditioned for cocaine compared to the saline control group (Fig. 5K). These results indicate that reduced levels of miR-124 in the NAc occur in animals that display cocaine-induced behavioral effects.

## Discussion

miRNAs are small (~ 17 to 24 nucleotide) single stranded RNAs that post-transcriptionally regulate gene expression^1^. miRNAs bind to the 3′ UTR of target mRNA and negatively regulate protein expression by translational repression^1,2,56^ or by mRNA degradation^57,58^. Since miRNAs can interact with multiple target mRNAs, these small RNAs regulate multiple cellular pathways^1,59,60^. Therefore, miRNAs are involved in almost every aspect of cellular biology^2,3^ including the regulation of normal brain function and neurological and psychiatric disorders^4^.

Accumulating evidence show that a growing number of miRNAs regulate protein expression in the CNS^4^. Specifically, miR-124 is emerging as a critical regulator of CNS function^5–7^. miR-124 accounts for ~ 25 percent of the total miRNA population in the brain and plays critical roles in the normal function and disease conditions of the CNS^19,61–64^. Importantly, recent studies indicate that miR-124 is linked to substance-use disorders including cocaine-seeking behavior and reward^12,17^. Consistent with these reports, we observed that acute or chronic exposure to cocaine downregulates miR-124 expression in the NAc of mouse brain (Fig. 4E,F) and in a dopaminergic neuronal cell model (Fig. 4A,B). Interestingly, miR-124 expression remained lower in the NAc when animals were exposed daily to cocaine for up to 6 days (Fig. 5A–C). Surprisingly, both acute and chronic cocaine treatment resulted in the induction of miR-124 expression in the VTA (Fig. 5H,I), but showed minimal effect in the PFC (Fig. 5D–F). Since cocaine exposure influences the NAc, VTA and PFC regions of the brain in distinct ways^65^, these results suggest that downregulation of miR-124 by cocaine is specific to the NAc.

The region-specific alterations in miR-124 expression (Figs. 4 and 5) are consistent regulation of a number of other miRNAs by cocaine in specific brain reward regions^12,14,66^. These findings are also in accordance with our recent report demonstrating downregulation of miR-125b expression in a region-specific manner of rodent brain upon cocaine exposure^48^. These region-specific changes in miRNA expression can manifest a variety of neuronal dysfunctions including dendritic morphology and long-lasting cocaine-mediated synaptic plasticity^67–69^. Alterations in structure and function of medium spiny neurons (MSNs) and their inputs during cocaine exposure^70,71^ are also dependent on localized gene/protein expression^67^. Therefore, it is plausible that cocaine-induced miR-124 regulation in specific brain regions may coordinate regionally-selective gene expression that drives behavioral changes during drug reward and addiction^65^. This prediction is supported by reduction in miR-124 levels in the NAc of animals conditioned for cocaine preference (Fig. 5K) and downregulation of the miR during cocaine-induced synaptic plasticity and behavioral changes^17^.

Identification of the neuronal genes regulated by miR-124 is critical to define the role of this key cellular miRNA in cocaine addiction and reward. miR-124 has been described to regulate several genes involved in neuronal function^72,73^. For example, miR-124 regulates genes involved in neurogenesis including *Scp1,* *Baf53a,* *Ptbp1,* *RhoG,* *Jagged1*, and *Sox9*^19^. Additionally, miR-124 regulates genes involved in glutamate signaling such as *Nr3c1* and *Gria4* ^29^ and the plasticity genes *Bdnf* and *Drd3*^17^. Interestingly, miR-124 has also been reported to suppress a network of non-neuronal genes involved in neuronal identity^23,24^. Our studies identified that miR-124 post-transcriptionally regulates PARP-1 expression in neuronal-like cells. First, our in silico studies utilized two algorithms to enhance confidence in the target predictions. RNAhybrid analysis employs a two-step process involving recognition of specific sequences of the mRNA and the formation of a stable secondary structure of the duplex^45,46^. Whereas, BiFold:RNA structures includes intramolecular base pairing between miR-124 and *Parp-1* 3′UTR in the prediction^47^. These analyses predicted a highly conserved binding site of miR-124 within the *Parp-1* 3′UTR and suggested a thermodynamically stable hair-pin loop structure formed between the two RNA molecules that is necessary for post-transcriptional regulation (Fig. 1A,B). Subsequently, we demonstrated through genetic and molecular studies a negative association between miR-124 levels and PARP-1 expression in a neuronal cell model (Fig. 1D–G). Interaction between miR-124 and 3′UTR of *Parp-1* was also confirmed by pull-down and mutational studies (Fig. 2). Collectively, these results established that miR-124 physically interacts with the *Parp-1* 3′UTR and post-transcriptionally regulates *Parp-1* in neuronal cells.

*Parp-1* can be regulated by both transcriptional and post-transcriptional mechanisms. Transcriptional regulation of *Parp-1* has been reported by factors such as Sp1, YY1, AP-2, Ets, and NF-kB^74–77^. Accordingly, in cancer cells, post-transcriptional regulations of *Parp-1* has been described by cellular miRNAs^78,79^. Previously, we have reported that miR-125b post-transcriptionally regulates PARP-1 by directly targeting the 3′UTR region^48^. Interesting, the seed sequence of miR-124 within the *Parp-1* 3′UTR is located in close proximity (10 nt upstream) to the seed sequence of miR-125b (Fig. 3). These observations suggested that these two cocaine-regulated miRNAs can both post-transcriptionally regulate *Parp-1*. Indeed, titration of these two miRs against each other confirmed that both miR-124 and miR-125b are post-transcriptional regulators of *Parp-1*. However, these studies illustrated that miR-124-mediated post-transcriptional regulation of *Parp-1* is stronger compared to the regulatory effect of miR-125b in vitro (Fig. 4). However, these data do not explain the mechanism by which miR-124 confers strong regulatory effects on *Parp-1*. Further studies are also warranted to examine whether miR-124 plays a dominant role in neuronal gene expression during cocaine exposure.

Cocaine-induced downregulation of miR-124 and identification of PARP-1 as a direct target of miR-124 highlight activation of a novel pathway during cocaine exposure. PARP-1 is a nuclear enzyme that plays critical roles during DNA damage and repair^30,33,34^. PARP-1 also functions as a transcriptional co-regulator by PARylating chromatin-binding proteins^30–34^. Specifically, linker histone H1 is one of the essential substrates of PARP-1 enzymatic activity whose regulation is critical for neuronal depolarization-dependent gene expression^80,81^. PARP-1 activity mediates H1 release from chromatin regions in depolarized post mitotic neurons along with significant accumulation of PARP-1 at CREB-related (*c-Fos,* *c-Jun,* *Egr-1*) promoters^82^. Importantly, PARP-1 is involved in the molecular effects of cocaine in the NAc^42–44^. Therefore, our results establishing miR-124 as a post-transcriptional regulator of PARP-1 in a neuronal cell model is highly significant since it provides important insight into the cellular and molecular effects of cocaine.

In summary, in this report we have: a) demonstrated down-regulation of the brain-enriched miRNA “miR-124” in a dopaminergic neuronal cell model and in the NAc, the major reward region of rodent brain, upon cocaine exposure, b) identified *Parp-1* *3′UTR* as a direct target of miR-124, and c) uncovered a post-transcriptional mechanism of regulation of PARP-1, a known regulator of cocaine action.

## Materials and methods

All the methods used in this study are in accordance with relevant guidelines and regulations.

### Reagents and chemicals

Cocaine hydrochloride (PubChem Substance ID 329775099), all-trans-retinoic acid (ATRA), antibodies of GAPDH and β actin were obtained from Sigma-Aldrich Chemicals (St. Louis, MO, USA). Antibodies to PARP-1 were purchased from Cell Signaling Technology (Danvers, MA, USA), whereas anti-dopamine transporter and anti-tyrosine hydroxylase were acquired from Abcam (Cambridge, MA, USA).

### In silico studies

To prediction hsa-miR-124 binding sites, the miR-124 sequences and the 3′ UTR of *Parp-1* gene were queried to two independent web platforms: RNAhybrid (version 2.1)^45,46^ and RNA Structures-BiFold^47^. RNAhybrid generates secondary structures based primarily on Minimum Free Energy (MFE). RNA Structures-BiFold algorithm considers intramolecular base pairings involved in the secondary structure formation. The putative targets were further analyzed using multiple sequence alignment-Clustal Omega tool. The sequence with the highest identity (> 90%) was chosen for further analysis in this study. For predicting the effects of nucleotide mutations on the secondary structure of miR-124 and *Parp-1* UTR we used RNAhybrid 2.1 and Bifold- RNAStructures (version 6.0.1; Mathews Lab; https://rna.urmc.rochester.edu/RNAstructureWeb/Servers/bifold/bifold.html)) web tools. Mutations performed on the *Parp-1* 3′UTR included base substitutions and base deletion in the predicted seed sequence region.

### Cell culture and cocaine treatment

Human neuroblastoma cells (SH-SY5Y) were purchased from American Type Culture Collection (Manassas, VA). The cells were maintained in a 1:1 mixture of DMEM and Ham’s F12 medium (Gibco, USA) supplemented with 10% (v/v) heat-inactivated fetal calf serum (Gibco) containing 2 mM glutamine and 1% antibiotics (penicillin–streptomycin). Cells were maintained and cultured at 37 °C in a humidified 5% CO_2_ atmosphere. SH-SY5Y were differentiated with ATRA as per published methods^48^. To mimic the physiologically relevant levels, cells treated with cocaine in a dose dependent manner from 1–100 μM to mimic physiologically relevant levels^83–88^. For acute treatment conditions, cells were treated with cocaine overnight for 16-18 h, whereas for chronic treatment, cells were exposed to cocaine with one treatment (50 μM) daily at a recurring interval of 24 h for five days.

### Animal studies

All animal use protocols were approved by the Mount Sinai Institutional Animal Care and Use Committee^48^. To study the effects of cocaine on miR-124, 9- to 11-wk-old C57BL/6 J male mice (The Jackson Laboratory, USA) were group-housed (five/cage) in a colony room set at a constant room temperature of 23 °C on a 12 h light/dark cycle (with lights kept on from 7:00 AM to 7:00 PM) with ad libitum access to food and water. For acute cocaine treatment experiments, the animals were injected with cocaine at 20 mg/kg body weight for one day whereas for chronic cocaine treatment conditions, the animals received daily intraperitoneal (i.p.) injections for seven consecutive days of cocaine at 20 mg/kg^48^. Control mice for all the experimental groups received saline injections. Bilateral 14-gauge NAc, PFc and VTA punches were taken from each animal and frozen immediately for RNA and protein studies^48^.

### Quantitative PCR analysis

SH-SY5Y cells were treated with cocaine and total RNA (including small RNA) was isolated using miRNeasy mini Kit (Qiagen) as per manufacturer’s instructions. For miR-124 amplification, cDNA was synthesized from the isolated total RNA using the Exiqon miRCURY LNA Universal microRNA- RT PCR. cDNA obtained was used as a template in the qPCR reaction mixture and PCR was performed using miRCURY LNA Syber Green mix (Exiqon) as per published methods^48^. The expression level (Ct values) of miR-124 was normalized to expression levels of 5 s rRNA as deltaCt values. For measuring *Parp-1* mRNA expression, cDNA synthesis was carried out with 100 ng of total RNA using iScript cDNA synthesis kit (Biorad, USA). qPCR analysis was performed with 50 ng of cDNA using iTaq Universal Sybr Green Super mix (Biorad, USA) using *Parp-1* specific primers [Forward: 5′-GAGGTGGATGGGTTCTCTGA-3′ and Reverse: 5′-ACACCCCTTGCACGTACTTC-3′] and *GAPDH* primers [Forward: 5′-GAAGGTGAAGGTCGGAGTC-3′ and Reverse: 5′-GAAGATGGTGATGGGATTTC-3′] as previously described (46). The expression level of *Parp-1* mRNA obtained was normalized to that of *GAPDH* mRNA expression.

To analyze miRNA levels in tissue samples, total RNA, including miRNAs, were extracted from NAc, PFc and VTA punches using All-in-One kit (Norgen Biotek, Thorold, Canada) as per the manufacturer’s instructions. The total RNA was eluted in 25 μL of nuclease free water from which 100 ng of total RNA was used for cDNA synthesis followed by qPCR analyses. All qPCR reactions based on Sybr green chemistry were run in triplicates on a 96 well plate with 1 µl of cDNA as template in a final reaction volume of 10 µl per well for each sample. For saline and cocaine-treated samples, the relative expression levels of miR-124 or, *Parp-1* were expressed as 2-Δ Ct values by comparing the respective ΔCt values obtained using *5srRNA* or *Gapdh* as normalizing targets. Fold change in miR-124 and *Parp-1* levels was calculated by comparing the 2-Δ Ct values of the cocaine-treated sample with that of respective (saline) control.

### Immunoblot analysis

SH-SY5Y cells were treated with appropriate concentrations of cocaine for 24 h, and then were washed with ice cold phosphate buffered saline (1X PBS, pH 7.2) and harvested by gentle scraping. Cell lysates were prepared using RIPA lysis and extraction buffer (Thermo Fisher Scientific) containing appropriate amounts of protease inhibitor and quantified according to standard BCA protein assay (Pierce, USA). Equal amounts of cell lysates were resolved on SDS–polyacrylamide gels and transferred to nitrocellulose membranes using a semi-dry blotter (Bio-Rad) and subjected to immunoblot with the primary antibodies for PARP-1 (1:1000) in blocking buffer (5% w/v non-fat milk in TBS, pH8.0; Sigma), and subsequently by a secondary antibody conjugated to horseradish peroxidase (1:5000). To determine GAPDH/β-actin expression blots were first stripped using Restore Plus stripping buffer (Pierce) at room temperature for 15–20 min then blocked and washed followed by overnight incubation with primary monoclonal antibodies for GAPDH or, β-actin at 1:2000 prepared in blocking buffer (5% w/v non-fat milk in TBS, pH8.0; Sigma). Densitometry analysis was performed by ImageStudio Digits version 5.2software (LI-COR, USA). Data analysis was performed by normalizing the expression intensities of PARP-1 to that of GAPDH/β-actin as housekeeping control.

### Knockdown and overexpression of miR-124

miR-124 inhibitors/mimics and scrambled controls were purchased from GE-Dharmacon (Lafayette, CO). 100–150 picomol (pM) of anti-miRs/miRNA mimics or scrambled controls were transfected into differentiated SH-SY5Y cells (4 × 10^5^ cells/well) grown in a 6 well culture plate using Lipofectamine 3,000 (Invitrogen) as per manufacturer’s protocol. Post transfection, cells were incubated for 36 h at 37 °C/5% CO2. Thereafter, cells were gently washed with phosphate-buffered saline (PBS), scraped, aliquoted for RNA and protein extraction, pelleted by centrifugation at 500 g for 5 min. Then the cell pellets were used for total RNA including miRNA and total protein isolation.

### Luciferase reporter assay

RFP tagged reporter construct containing the full-length (785 nt) 3′-UTR fragment of the *Parp-1* gene (accession number: NM_001618.3) containing the target sequence for miR-124 was cloned downstream of the luciferase gene in the pmiRTarget vector (OriGene Technologies, Rockville, MD). The control plasmid (pPARP-Null) without the *Parp-1* 3′UTR sequence was generated by restriction digestion of the full-length plasmid as described previously (46). Considering the results obtained from the initial in silico studies, we carried out site-directed mutagenesis using the Site-Directed Mutagenesis Kit (NEB, USA) following manufacturer’s protocol. For base substitutions mutations performed and generated were pPARP-1 mutant 1 (CC > GG), pPARP-1 mutant 2 (TG > CA), pPARP-1 mutant 3 (GC > AA). For deletion mutation, the mutant generated was pPARP-1 mutant ΔGC. Mutations were confirmed by sequencing.

Differentiated SH-SY5Y cells (5 × 10^4^–1 × 10^5^ cells/well) in a 24 well plate were transfected with either the control plasmid without *Parp-1* 3′UTR (pmiRTarget), plasmid harboring the full length *Parp-1* 3′UTR (pmiRTarget-UTR) and plasmids harboring the seed sequence mutations on *Parp-1* 3′UTR [(CC > GG), (TG > CA), (GC > AA) and (ΔGC)] independently in the presence or absence of miR-124 mimics and anti-miR124 using Lipofectamine 3000 (Invitrogen) and were incubated for 48 h. The transfection efficiency was assayed by RFP expression of the Luciferase reporter plasmid. Transfected cells after treatment were lysed using ice-cold 1X passive lysis buffer (Promega, USA) and luciferase activity of the cell extracts was measured using a plate reader (BioTek, USA). Samples were assayed in triplicate. Data are shown as relative luciferase activity normalized to the expression of RFP.

### Pull-down assay

3′-ends of both miR-124-3p (5′-UAAGGCACGCGGUGAAUGCCAA/3′Bio) and Scrambled oligonucleotide (5′-GAUGGCAUUCGAUCAGUUCUA/3′Bio) were custom modified with a biotin tag (Exiqon, Germany). Lyophilized oligonucleotides were reconstituted to a final concentration of 25 µM. For biotin pulldown experiments 75 pM of oligonucleotide (3′-biotin tagged) were transfected to differentiated SH-SY5Y cells and were incubated for 36 h. Cells were harvested by gentle scraping and were processed further for target validation experiments. The freshly prepared cellular lysates were incubated with the blocked streptavidin coated magnetic beads on a bench top nutating mixer for 1 h at room temperature. Following which the beads were processed and washed using freshly prepared ice- cold pull-down wash buffer on the magnetic separator. Finally, the beads were resuspended in 100 µL of nuclease free water. Half the volume of this reconstituted complex was processed for on-column DNAse digestion (Ambion, USA) followed by column purification and enrichment using Qiagen RNAeasy purification kit. Post purification, both the eluates from crude complex (unpurified beads) and purified complex were used as templates for cDNA synthesis using Biorad iScript Reverse Transcriptase kit.

To detect the mRNA targets of miR-124 from the pull-down reaction mixture, we employed qPCR measurements. We designed primers within the open reading frame (ORF) to amplify *Parp-1* mRNA. We also included primers sets for detecting *Ptbp-1* mRNA, a known target of miR-124b as a positive control ^52^ and ß-actin mRNA as a non-specific negative control. We also designed primers for detecting the 3′UTR regions of *Parp-1*, *Ptbp-1,* and *Actb* (ß-actin). In the cDNA synthesis reactions, 50 ng of column purified RNA and 1 µl of crude bead eluate were used as templates in separate reactions to perform cDNA synthesis using oligo dT primers to capture mRNAs associated with the miRNA complex. The cDNA generated from these reactions served as templates for qPCR reactions. All qPCR reactions were performed in triplicates in a Biorad 96 well clear bottom plate in sterile conditions as per published protocol ^48^. The expression levels of target genes were analyzed relative to their expression in scrambled controls. Data was plotted in fold change that was calculated by comparing the expression values of the target genes with that of scrambled control.

### Conditioned place preference (CPP)

CPP was conducted in mice as described previously with some modifications^55^. Briefly, three-chamber boxes consisted of a smaller middle chamber and two larger conditioning chambers with different contextual cues such as gray or striped walls with different floor meshes. During pre-conditioning (day 1, Pretest), mice were allowed to explore the three compartments freely for 20 min. The time spent in each chamber was recorded, and the mice were arranged into control group and cocaine group in an unbiased manner. Mice were conditioned for 30 min over 6 d to the saline-paired side in the morning and the cocaine-paired side (20 mg/kg i.p.) in the afternoon. On day 8 (Post-test), mice were placed again in the central compartment and allowed to move freely between the chambers for 20 min and the time spent in each chamber was recorded. CPP scores were calculated as time spent in the cocaine-paired chamber minus time spent in the saline-paired chamber. After the CPP test, mice were euthanized within 1 h and punches of the NAc were flash frozen on dry ice and stored for analyses.

### Statistical analysis

Data were expressed as mean ± SEM obtained from appropriate number of independent experiments conducted in triplicates, as stated. Statistical significance was assessed using GraphPad Prism software, version 8.2.1 (GraphPad Prism Software, La Jolla, CA, USA). Unpaired or paired two-tailed t-tests were used as stated to analyze data involving direct comparison of an experimental group with a control group. One- or two-way analysis of variance (ANOVA) was used with repeated measures for appropriate experimental groups as stated followed by Tukey’s and Bonferroni’s multiple comparison correction. For quantitative-PCR experiments, we performed unpaired two-tailed *t* test to determine the significance in target gene expression in control versus treated mice. For nucleic acid pulldown studies, two-way ANOVA by comparing the mean of each row across columns was performed to determine the enrichment of target genes. The reported p-values were adjusted to account for multiple comparisons. For all statistical tests, a two-sided confidence level of *p* < 0.05 (95% confidence interval) was accepted for statistical significance.

## Supplementary information

Supplementary information.
