# Supplementary figures and images for: Cocaine-regulated microRNA miR-124 controls poly (ADP-ribose) polymerase-1 expression in neuronal cells
Source: Sci Rep. 2020 Jul 8;10:11197. doi: 10.1038/s41598-020-68144-6 (PMC7343862; doi:10.1038/s41598-020-68144-6)

**Figure 1 F-G**  
**(Original Blots)**

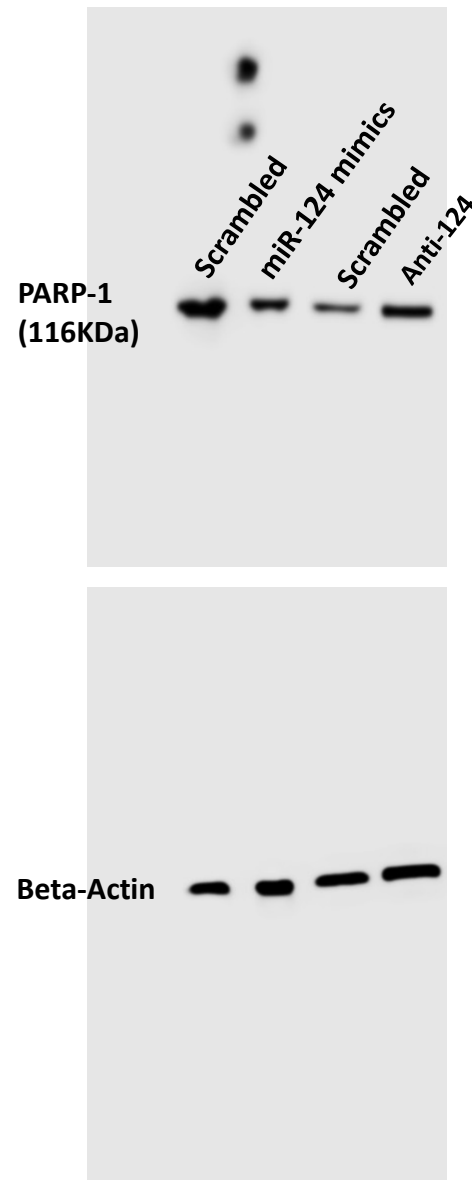

**Figure 4C**  
**(Original Blots)**

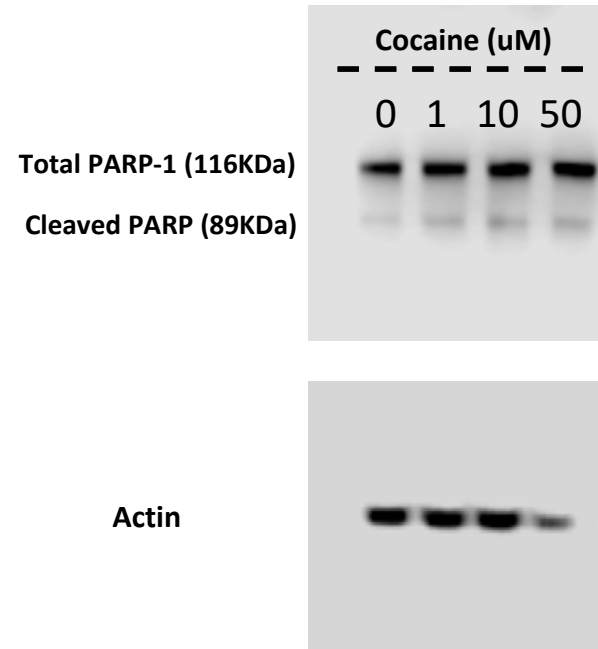

Supplement: Supplementary file 1 — Supplementary information. [file 41598_2020_68144_MOESM1_ESM.pdf]
